# Supplementary material for: Evaluating machine learning approaches for host prediction using H3 influenza genomic data
Source: PLoS One. 2025 Nov 5;20(11):e0336142. doi: 10.1371/journal.pone.0336142 (PMC12588535; doi:10.1371/journal.pone.0336142)
Supplement: S1 Table — Sequence labels included from each species class after extracting the hosts from the labels and preprocessing to only have the classes of canine, equine, goose, human, mallard, swine, duck, and environment. (DOCX) [file pone.0336142.s001.docx]

**S1 Table. Sequence labels included from each class.** Sequence labels included from each species class after extracting the hosts from the labels and preprocessing to only have the classes of canine, equine, goose, human, mallard, swine, duck, and environment.

| Class | Labels included |
| --- | --- |
| Canine | Canine, Canis lupus familiaris |
| Chicken | Chicken, Korean native chicken, Silkie chicken, Wild chicken |
| Equine | Equine, Equus caballus, Equus ferus caballus, Horse |
| Goose | Bar-headed goose (Anser indicus), Barnacle goose, Emperor goose, Goose, Greater white-fronted goose, Snow goose, Swan goose |
| Human | All available countries and cities |
| Mallard | Anas platyrhynchos, Mallard, Mallard-black duck hybrid, Mallard duck, Mallard (Anas platyrhynchos), Wild duck* |
| Swine | Swine, sus scrofa** |
| Duck | American black duck, American green-winged teal, American widgeon, Black duck, Black scoter, Blue-winged teal, Bucefala clangula, Bufflehead, Chestnut teal, Cinnamon teal, Common eider, Common teal, Domestic duck, Domestic green-winged teal, Duck, Eurasian teal, Gadwall, Gadwall duck, Garganey, Green-winged teal, Grey teal, Hooded merganser, Lesser scaup, Long-tailed duck, Muscovy duck, Northern pintail, Northern shoveler, Pacific black duck, Pink-eared duck, Pintail, Redhead, Ring-necked duck, Ruddy duck, Ruddy shelduck, Shoveler, Spectacled eider, Spot-billed duck, Teal, White peckin duck, Widgeon, Wood duck, Yellow-billed pintail |
| Environment | Environment, surface water***, water*** |

*6 sequence sets labelled as wild duck were included as mallard

**2 sequence sets labelled as sus scrofa were included as swine

***1 sequence set labelled as surface water and 1 sequence set labelled as water
