# Supplementary material for: Evaluating machine learning approaches for host prediction using H3 influenza genomic data
Source: PLoS One. 2025 Nov 5;20(11):e0336142. doi: 10.1371/journal.pone.0336142 (PMC12588535; doi:10.1371/journal.pone.0336142)
Supplement: S2 Table — Subtype distribution of the 21429 H3 whole genome sequence sets by host class retrieved from the NCBI Influenza Virus Database, BV-BRC database, and EpiFlu database after preprocessing was completed. (DOCX) [file pone.0336142.s002.docx]

**S2 Table. Subtype distribution after preprocessing.** Subtype distribution of the 21429 H3 whole genome sequence sets by host class retrieved from the NCBI Influenza Virus Database, BV-BRC database, and EpiFlu database after preprocessing was completed.

| Subtype | Human | Swine | Duck | Mallard | Chicken | Canine | Equine | Environment | Goose |
| --- | --- | --- | --- | --- | --- | --- | --- | --- | --- |
| H3 | 44 | 0 | 5 | 0 | 0 | 0 | 0 | 0 | 1 |
| H3N1 | 0 | 33 | 29 | 17 | 2 | 0 | 0 | 0 | 0 |
| H3N2 | 16515 | 2280 | 171 | 127 | 4 | 255 | 0 | 18 | 1 |
| H3N2v* | 1 | 0 | 0 | 0 | 0 | 0 | 0 | 0 | 0 |
| H3N3 | 1 | 2 | 10 | 6 | 0 | 0 | 0 | 0 | 2 |
| H3N4 | 0 | 0 | 0 | 0 | 0 | 0 | 0 | 0 | 0 |
| H3N5 | 0 | 0 | 5 | 9 | 0 | 0 | 0 | 0 | 0 |
| H3N6 | 0 | 1 | 89 | 57 | 0 | 6 | 0 | 35 | 3 |
| H3N7 | 0 | 0 | 6 | 1 | 0 | 0 | 0 | 0 | 8 |
| H3N8 | 5 | 2 | 658 | 488 | 255 | 33 | 168 | 52 | 16 |
| H3N9 | 0 | 0 | 4 | 8 | 0 | 0 | 0 | 0 | 0 |
| Total | 16566 | 2316 | 977 | 713 | 261 | 294 | 168 | 105 | 29 |
