# Supplementary material for: Evaluating machine learning approaches for host prediction using H3 influenza genomic data
Source: PLoS One. 2025 Nov 5;20(11):e0336142. doi: 10.1371/journal.pone.0336142 (PMC12588535; doi:10.1371/journal.pone.0336142)
Supplement: S3 Table — Top 10 features with the highest mean decrease Gini scores after feature selection for each of the 8 preliminary random forest models. (DOCX) [file pone.0336142.s003.docx]

**S3 Table. Top 10 most important features for each genome segment.** Top 10 features with the highest mean decrease Gini scores after feature selection for each of the 8 preliminary random forest models.

| Rank | Selected by HA model | Selected by NA model | Selected by NP model | Selected by PA model | Selected by PB2 model | Selected by PB1 model | Selected by NS model | Selected by MP model |
| --- | --- | --- | --- | --- | --- | --- | --- | --- |
| 1 | GCACG | GATAGT | AEI | NCR | PPE | TRN | EGP | TGGTGG |
| 2 | ACATGG | GGT | NSL | CGC | Net charge | TGTGGC | SKN | CTGACG |
| 3 | CACAGT | TATAG | GPA | LS | TSG | GACCTT | LLF* | FVN* |
| 4 | TGTTT | GTGT | SLT | ELD | ACTTGT | ITR | MTM | IAL |
| 5 | AGCACG | CTGAAG | GCCTTC | LDD | EH | GTGG | RNW | ATACGG |
| 6 | ATGCTT | ATAGTG | GRK | SC | YFE | GAAAG | GCTCAT | TGTGCA |
| 7 | AF | TGTG | LL | LRS | HID | KNQ | TGTTTG | Q* |
| 8 | GCTTTT | TATGAA | GGE | GTCA | ATGAT | ATCTTT | FEV* | TTAAGA |
| 9 | CATGTT | TTCCGC | MRT | LLS | FD | KN | AGGTCA | GTTGAC |
| 10 | CATGT | GCTGC | PAV | AS | FE | AGTGG | MS | GGATGT |

*Feature from protein 2 in models that had two proteins (PB1, NS, MP)
