# Supplementary material for: Evaluating machine learning approaches for host prediction using H3 influenza genomic data
Source: PLoS One. 2025 Nov 5;20(11):e0336142. doi: 10.1371/journal.pone.0336142 (PMC12588535; doi:10.1371/journal.pone.0336142)
Supplement: S4 Table — F1 scores for each of the 7 classes of canine, chicken, equine, goose, human, mallard, and swine obtained during model validation using the trained models from each genome segment on their respective test datasets. (DOCX) [file pone.0336142.s004.docx]

**S4 Table. F1 Scores for each class on the testing dataset.** F1 scores for each of the 7 classes of canine, chicken, equine, goose, human, mallard, and swine obtained during model validation using the trained models from each genome segment on their respective test datasets.

| Segment | Canine | Chicken | Equine | Goose | Human | Mallard | Swine | Macro F1 |
| --- | --- | --- | --- | --- | --- | --- | --- | --- |
| **HA Models** |  |  |  |  |  |  |  |  |
| RF | 1.000 | 0.9673 | 1.000 | 0.5456 | 0.9988 | 0.9748 | 0.9904 | 0.9252 |
| XGB | 1.000 | 0.9673 | 1.000 | 0.6154 | 0.9987 | 0.9769 | 0.9904 | 0.9355 |
| Ridge | 1.000 | 0.9673 | 1.000 | 0 | 0.9984 | 0.9682 | 0.9874 | 0.8459 |
| **NA Models** |  |  |  |  |  |  |  |  |
| RF | 1.000 | 0.9673 | 1.000 | 0.6667 | 0.9988 | 0.9771 | 0.9904 | 0.9429 |
| XGB | 1.000 | 0.9673 | 1.000 | 0.6667 | 0.9989 | 0.9771 | 0.9911 | 0.9430 |
| Ridge | 1.000 | 0.9673 | 1.000 | 0.2222 | 0.9986 | 0.9680 | 0.9881 | 0.8778 |
| **NP Models** |  |  |  |  |  |  |  |  |
| RF | 1.000 | 0.9610 | 1.000 | 0.7692 | 0.9983 | 0.9792 | 0.9874 | 0.9565 |
| XGB | 1.000 | 0.9548 | 1.000 | 0.6667 | 0.9985 | 0.9770 | 0.9882 | 0.9407 |
| Ridge | 1.000 | 0.9673 | 1.000 | 0.2222 | 0.9983 | 0.9780 | 0.9874 | 0.8776 |
| **PA Models** |  |  |  |  |  |  |  |  |
| RF | 0.9935 | 0.9673 | 1.000 | 0.8571 | 0.9985 | 0.9770 | 0.9889 | 0.9689 |
| XGB | 0.9935 | 0.9673 | 1.000 | 0.8571 | 0.9989 | 0.9793 | 0.9911 | 0.9696 |
| Ridge | 0.9935 | 0.9673 | 1.000 | 0.2222 | 0.9985 | 0.9682 | 0.9882 | 0.8768 |
| **PB2 Models** |  |  |  |  |  |  |  |  |
| RF | 0.9935 | 0.9673 | 1.000 | 0.8571 | 0.9986 | 0.9792 | 0.9889 | 0.9692 |
| XGB | 0.9935 | 0.9610 | 1.000 | 0.8561 | 0.9985 | 0.9838 | 0.9889 | 0.9690 |
| Ridge | 0.9935 | 0.9673 | 1.000 | 0.5455 | 0.9986 | 0.9748 | 0.9882 | 0.9240 |
| **PB1 Models** |  |  |  |  |  |  |  |  |
| RF | 1.000 | 0.9673 | 1.000 | 0.5455 | 0.9987 | 0.9748 | 0.9896 | 0.9251 |
| XGB | 1.000 | 0.9673 | 1.000 | 0.6154 | 0.9987 | 0.9747 | 0.9896 | 0.9351 |
| Ridge | 1.000 | 0.9610 | 1.000 | 0 | 0.9987 | 0.9704 | 0.9896 | 0.8457 |
| **NS Models** |  |  |  |  |  |  |  |  |
| RF | 1.000 | 0.9673 | 1.000 | 0.7692 | 0.9986 | 0.9771 | 0.9896 | 0.9574 |
| XGB | 1.000 | 0.9737 | 1.000 | 0.6667 | 0.9986 | 0.9725 | 0.9896 | 0.9430 |
| Ridge | 1.000 | 0.9673 | 1.000 | 0.4000 | 0.9985 | 0.9704 | 0.9889 | 0.9036 |
| **MP Models** |  |  |  |  |  |  |  |  |
| RF | 0.9935 | 0.9673 | 1.000 | 0.6154 | 0.9982 | 0.9700 | 0.9867 | 0.9330 |
| XGB | 0.9935 | 0.9737 | 1.000 | 0.6154 | 0.9982 | 0.9700 | 0.9860 | 0.9338 |
| Ridge | 0.9935 | 0.9673 | 1.000 | 0.3636 | 0.9984 | 0.9703 | 0.9867 | 0.8971 |
