# Supplementary material for: Evaluating machine learning approaches for host prediction using H3 influenza genomic data
Source: PLoS One. 2025 Nov 5;20(11):e0336142. doi: 10.1371/journal.pone.0336142 (PMC12588535; doi:10.1371/journal.pone.0336142)
Supplement: S5 Table — Class specificities for each of the 7 classes of canine, chicken, equine, goose, human, mallard, and swine obtained during model validation using the trained models from each genome segment on their respective test datasets. (DOCX) [file pone.0336142.s005.docx]

**S5 Table. Class specificities for each class on the testing dataset.** Class specificities for each of the 7 classes of canine, chicken, equine, goose, human, mallard, and swine obtained during model validation using the trained models from each genome segment on their respective test datasets.

| Segment | Canine | Chicken | Equine | Goose | Human | Mallard | Swine |
| --- | --- | --- | --- | --- | --- | --- | --- |
| **HA Models** |  |  |  |  |  |  |  |
| RF | 1.000 | 0.9998 | 1.000 | 1.000 | 0.9901 | 0.9981 | 1.000 |
| XGB | 1.000 | 0.9998 | 1.000 | 0.9998 | 0.9892 | 0.9985 | 1.000 |
| Ridge | 1.000 | 0.9998 | 1.000 | 1.000 | 0.9865 | 0.9976 | 1.000 |
| **NA Models** |  |  |  |  |  |  |  |
| RF | 1.000 | 0.9998 | 1.000 | 1.000 | 0.9901 | 0.9983 | 1.000 |
| XGB | 1.000 | 0.9998 | 1.000 | 1.000 | 0.9910 | 0.9983 | 1.000 |
| Ridge | 1.000 | 0.9998 | 1.000 | 1.000 | 0.9883 | 0.9978 | 0.9998 |
| **NP Models** |  |  |  |  |  |  |  |
| RF | 1.000 | 0.9997 | 1.000 | 1.000 | 0.9883 | 0.9986 | 0.9994 |
| XGB | 1.000 | 0.9995 | 1.000 | 1.000 | 0.9901 | 0.9985 | 0.9994 |
| Ridge | 1.000 | 0.9998 | 1.000 | 1.000 | 0.9883 | 0.9978 | 0.9994 |
| **PA Models** |  |  |  |  |  |  |  |
| RF | 1.000 | 0.9998 | 1.000 | 1.000 | 0.9874 | 0.9985 | 1.000 |
| XGB | 1.000 | 0.9998 | 1.000 | 1.000 | 0.9910 | 0.9985 | 1.000 |
| Ridge | 1.000 | 0.9998 | 1.000 | 1.000 | 0.9892 | 0.9976 | 0.9996 |
| **PB2 Models** |  |  |  |  |  |  |  |
| RF | 1.000 | 0.9998 | 1.000 | 1.000 | 0.9883 | 0.9986 | 0.9998 |
| XGB | 1.000 | 0.9997 | 1.000 | 1.000 | 0.9874 | 0.9990 | 0.9998 |
| Ridge | 1.000 | 0.9998 | 1.000 | 1.000 | 0.9892 | 0.9981 | 0.9996 |
| **PB1 Models** |  |  |  |  |  |  |  |
| RF | 1.000 | 0.9998 | 1.000 | 1.000 | 0.9892 | 0.9981 | 1.000 |
| XGB | 1.000 | 0.9998 | 1.000 | 0.9998 | 0.9892 | 0.9983 | 1.000 |
| Ridge | 1.000 | 0.9997 | 1.000 | 1.000 | 0.9892 | 0.9978 | 1.000 |
| **NS Models** |  |  |  |  |  |  |  |
| RF | 1.000 | 0.9998 | 1.000 | 1.000 | 0.9892 | 0.9983 | 1.000 |
| XGB | 1.000 | 1.000 | 1.000 | 1.000 | 0.9883 | 0.9981 | 1.000 |
| Ridge | 1.000 | 0.9998 | 1.000 | 1.000 | 0.9892 | 0.9978 | 0.9998 |
| **MP Models** |  |  |  |  |  |  |  |
| RF | 1.000 | 0.9998 | 1.000 | 0.9998 | 0.9874 | 0.9983 | 0.9993 |
| XGB | 1.000 | 1.000 | 1.000 | 0.9998 | 0.9874 | 0.9983 | 0.9991 |
| Ridge | 1.000 | 0.9998 | 1.000 | 0.9998 | 0.9892 | 0.9980 | 0.9993 |
