# Supplementary material for: Evaluating machine learning approaches for host prediction using H3 influenza genomic data
Source: PLoS One. 2025 Nov 5;20(11):e0336142. doi: 10.1371/journal.pone.0336142 (PMC12588535; doi:10.1371/journal.pone.0336142)
Supplement: S6 Table — Segment host prediction counts obtained from using the best performing models per genome segment (random forest model for the NS segment, XGBoost models for the remaining 7 segments) on the test dataset of 6078 sequences. (DOCX) [file pone.0336142.s006.docx]

**S6 Table. Segment host prediction counts on the testing dataset.** Segment host prediction counts obtained from using the best performing models per genome segment (random forest model for the NS segment, XGBoost models for the remaining 7 segments) on the test dataset of 6078 sequences

| Scenario | Count | % |
| --- | --- | --- |
| Correct prediction (e.g. human host, 8 human) | 6043 | 99.4 |
| Correct but mixed host prediction (e.g. human host, 7 human, 1 swine) | 13 | 0.213 |
| Misclassified and all segments predict the same host (e.g. human host, 8 swine) | 15 | 0.247 |
| Misclassified and mixed host prediction (e.g. human host, 7 canine, 1 human) | 7 | 0.115 |
| Total | 6078 |  |
