# Supplementary material for: Evaluating machine learning approaches for host prediction using H3 influenza genomic data
Source: PLoS One. 2025 Nov 5;20(11):e0336142. doi: 10.1371/journal.pone.0336142 (PMC12588535; doi:10.1371/journal.pone.0336142)
Supplement: S7 Table — (DOCX) [file pone.0336142.s007.docx]

**S7 Table. Accession numbers and labels for misclassified PB2 human environment sequences.**

| Accession # | Label |
| --- | --- |
| CY146753 | A/environment/Hunan/S4350/2011 (H3N8) |
| OR048386 | A/environment/Japan/KU-B11/2020 (H3N6) |
| OR044163 | A/environment/Japan/KU-B14/2020 (H3N8) |
| OR048410 | A/environment/Japan/KU-B8/2020 (H3N2) |
| OR044179 | A/environment/Japan/KU-C10/2020 (H3N8) |
| OR044195 | A/environment/Japan/KU-D10/2020 (H3N8) |
| OR048458 | A/environment/Japan/KU-D9/2020 (H3N2) |
| OR048602 | A/environment/Japan/KU-I13/2020 (H3N2) |
| OR048666 | A/environment/Japan/KU-J9/2020 (H3N2) |
|  |  |
