# Supplementary material for: Evaluating machine learning approaches for host prediction using H3 influenza genomic data
Source: PLoS One. 2025 Nov 5;20(11):e0336142. doi: 10.1371/journal.pone.0336142 (PMC12588535; doi:10.1371/journal.pone.0336142)
Supplement: S8 Table — Mean, median, mode, minimum, and maximum patristic distances were calculated from the representative HA maximum-likelihood phylogenetic tree. (DOCX) [file pone.0336142.s008.docx]

**S8 Table. Summary statistics for patristic distances from the maximum-likelihood phylogenetic tree.** Mean, median, mode, minimum, and maximum patristic distances were calculated from the representative HA maximum-likelihood phylogenetic tree.

| Summary Statistics | Pairwise Distance Value |
| --- | --- |
| Mean | 0.301 |
| Median | 0.318 |
| Mode | N/A |
| Min | 0.002^1^ |
| Max | 0.567 |

^1^Representative sequences were obtained for each class and case study separately to ensure that at least one representative was present, therefore it is possible for two of the representative sequences to be nearly identical to each other
