# Supplementary material for: Evaluating machine learning approaches for host prediction using H3 influenza genomic data
Source: PLoS One. 2025 Nov 5;20(11):e0336142. doi: 10.1371/journal.pone.0336142 (PMC12588535; doi:10.1371/journal.pone.0336142)
Supplement: S9 Table — Mean, median, and patristic distance range for each of the species-species pairs. (DOCX) [file pone.0336142.s009.docx]

**S9 Table. Summary statistics for species-species patristic distances.** Mean, median, and patristic distance range for each of the species-species pairs.

| Host-Pair | Type | Number of Pairs | Mean Pairwise Distance | Median Pairwise Distance | Pairwise Distance Range |
| --- | --- | --- | --- | --- | --- |
| Canine-Canine | within | 1 | 0.45887274 | 0.45887274 | 0.459 - 0.459 |
| Canine-Chicken | between | 8 | 0.31967998 | 0.35879053 | 0.105 - 0.493 |
| Canine-Duck | between | 32 | 0.32844203 | 0.36697583 | 0.105 - 0.468 |
| Canine-Environment | between | 16 | 0.37494334 | 0.39167941 | 0.138 - 0.558 |
| Canine-Equine | between | 4 | 0.26676058 | 0.28162574 | 0.048 - 0.456 |
| Canine-Goose | between | 6 | 0.34585688 | 0.37370468 | 0.153 - 0.427 |
| Canine-Human | between | 10 | 0.37619929 | 0.39247252 | 0.144 - 0.541 |
| Canine-Mallard | between | 16 | 0.32032073 | 0.36680928 | 0.08 - 0.442 |
| Canine-Swine | between | 28 | 0.40060988 | 0.39739986 | 0.062 - 0.567 |
| Chicken-Chicken | within | 6 | 0.24843701 | 0.25159124 | 0.132 - 0.352 |
| Chicken-Duck | between | 64 | 0.23630042 | 0.24042274 | 0.003 - 0.433 |
| Chicken-Environment | between | 32 | 0.29674578 | 0.33172941 | 0.046 - 0.434 |
| Chicken-Equine | between | 8 | 0.42595858 | 0.4231695 | 0.368 - 0.49 |
| Chicken-Goose | between | 12 | 0.23925623 | 0.26601845 | 0.043 - 0.363 |
| Chicken-Human | between | 20 | 0.27079096 | 0.28426596 | 0.013 - 0.401 |
| Chicken-Mallard | between | 32 | 0.21924555 | 0.22440899 | 0.049 - 0.381 |
| Chicken-Swine | between | 56 | 0.33051827 | 0.35515452 | 0.052 - 0.465 |
| Duck-Duck | within | 120 | 0.26037213 | 0.25452979 | 0.116 - 0.408 |
| Duck-Environment | between | 128 | 0.3010799 | 0.32957903 | 0.002 - 0.498 |
| Duck-Equine | between | 32 | 0.40732252 | 0.41548099 | 0.196 - 0.465 |
| Duck-Goose | between | 48 | 0.25180312 | 0.28401243 | 0.033 - 0.367 |
| Duck-Human | between | 80 | 0.28892368 | 0.29849355 | 0.04 - 0.481 |
| Duck-Mallard | between | 128 | 0.23312051 | 0.22904827 | 0.016 - 0.383 |
| Duck-Swine | between | 224 | 0.33275039 | 0.35325399 | 0.061 - 0.507 |
| Environment-Environment | within | 28 | 0.34984046 | 0.35750802 | 0.146 - 0.499 |
| Environment-Equine | between | 16 | 0.42007772 | 0.42350713 | 0.197 - 0.556 |
| Environment-Goose | between | 24 | 0.28486445 | 0.30796735 | 0.033 - 0.429 |
| Environment-Human | between | 40 | 0.30105398 | 0.32575346 | 0.023 - 0.482 |
| Environment-Mallard | between | 64 | 0.28494598 | 0.3147493 | 0.035 - 0.446 |
| Environment-Swine | between | 112 | 0.32919948 | 0.34936364 | 0.026 - 0.53 |
| Equine-Equine | within | 1 | 0.1327925 | 0.1327925 | 0.133 - 0.133 |
| Equine-Goose | between | 6 | 0.40641733 | 0.40559389 | 0.387 - 0.425 |
| Equine-Human | between | 10 | 0.46467404 | 0.47736476 | 0.388 - 0.538 |
| Equine-Mallard | between | 16 | 0.40692727 | 0.4071133 | 0.355 - 0.44 |
| Equine-Swine | between | 28 | 0.4608548 | 0.49866305 | 0.06 - 0.564 |
| Goose-Goose | within | 3 | 0.26028969 | 0.29078403 | 0.192 - 0.298 |
| Goose-Human | between | 15 | 0.3070846 | 0.32238723 | 0.036 - 0.412 |
| Goose-Mallard | between | 24 | 0.2261156 | 0.26260615 | 0.042 - 0.315 |
| Goose-Swine | between | 42 | 0.34756274 | 0.3598688 | 0.062 - 0.437 |
| Human-Human | within | 10 | 0.25333037 | 0.2477311 | 0.127 - 0.402 |
| Human-Mallard | between | 40 | 0.27758008 | 0.3020399 | 0.024 - 0.429 |
| Human-Swine | between | 70 | 0.24432881 | 0.24323986 | 0.024 - 0.513 |
| Mallard-Mallard | within | 28 | 0.23457412 | 0.2606203 | 0.127 - 0.329 |
| Mallard-Swine | between | 112 | 0.32376891 | 0.34018997 | 0.071 - 0.455 |
| Swine-Swine | within | 91 | 0.26560852 | 0.2636167 | 0.04 - 0.539 |
