# Supplementary figures and images for: Evaluating machine learning approaches for host prediction using H3 influenza genomic data
Source: PLoS One. 2025 Nov 5;20(11):e0336142. doi: 10.1371/journal.pone.0336142 (PMC12588535; doi:10.1371/journal.pone.0336142)

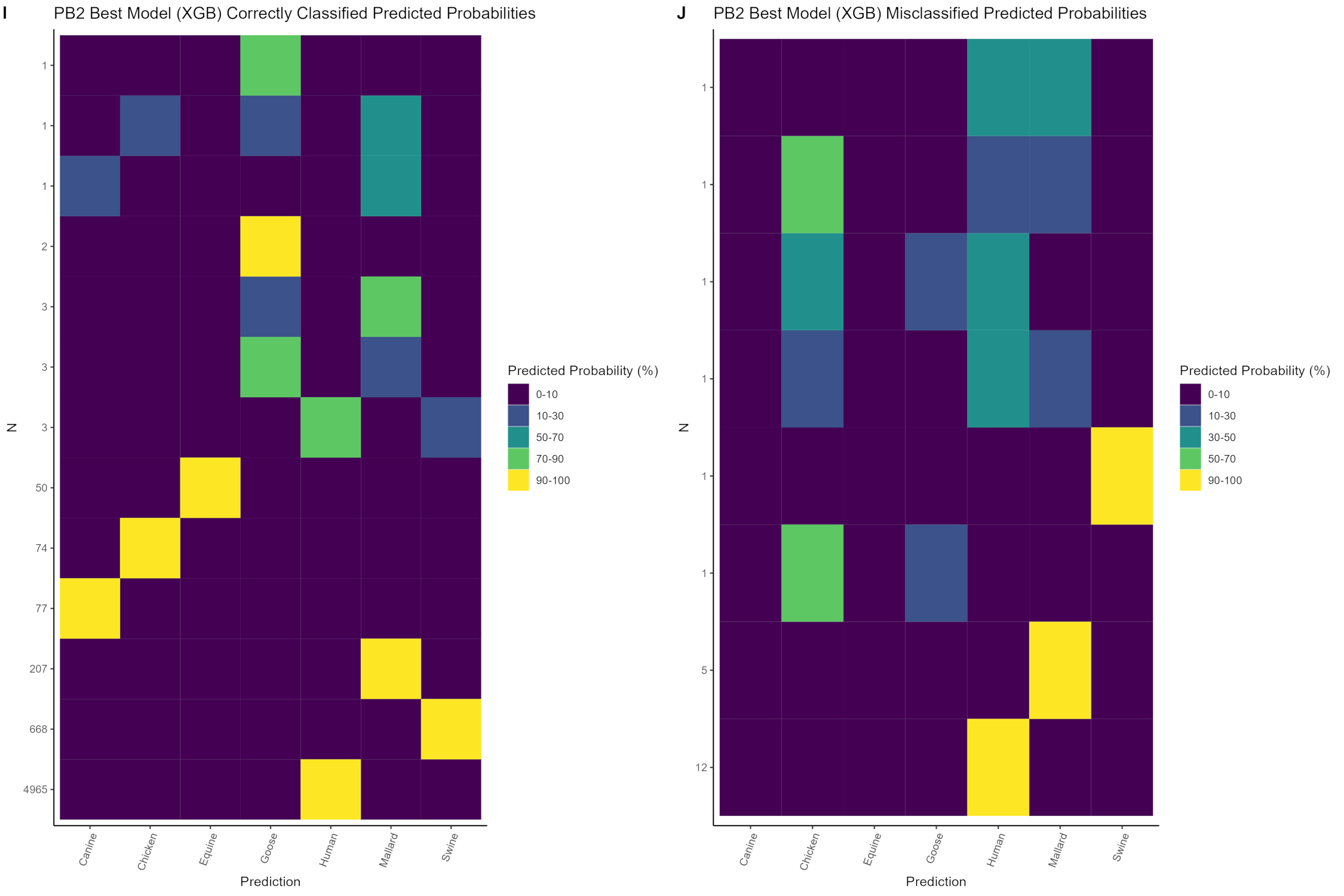

Supplement: S1 Fig — Heatmaps for the PB2 genome segment using the XGBoost model (XGB), A shows the predicted probabilities for the correctly classified sequences and B shows the predicted probabilities for the misclassified sequences. Predicted probabilities are read as rows, and the N on the y axis denotes the number of sequences with the predicted probability pattern shown for the respective row. (TIFF) [file pone.0336142.s010.tiff]

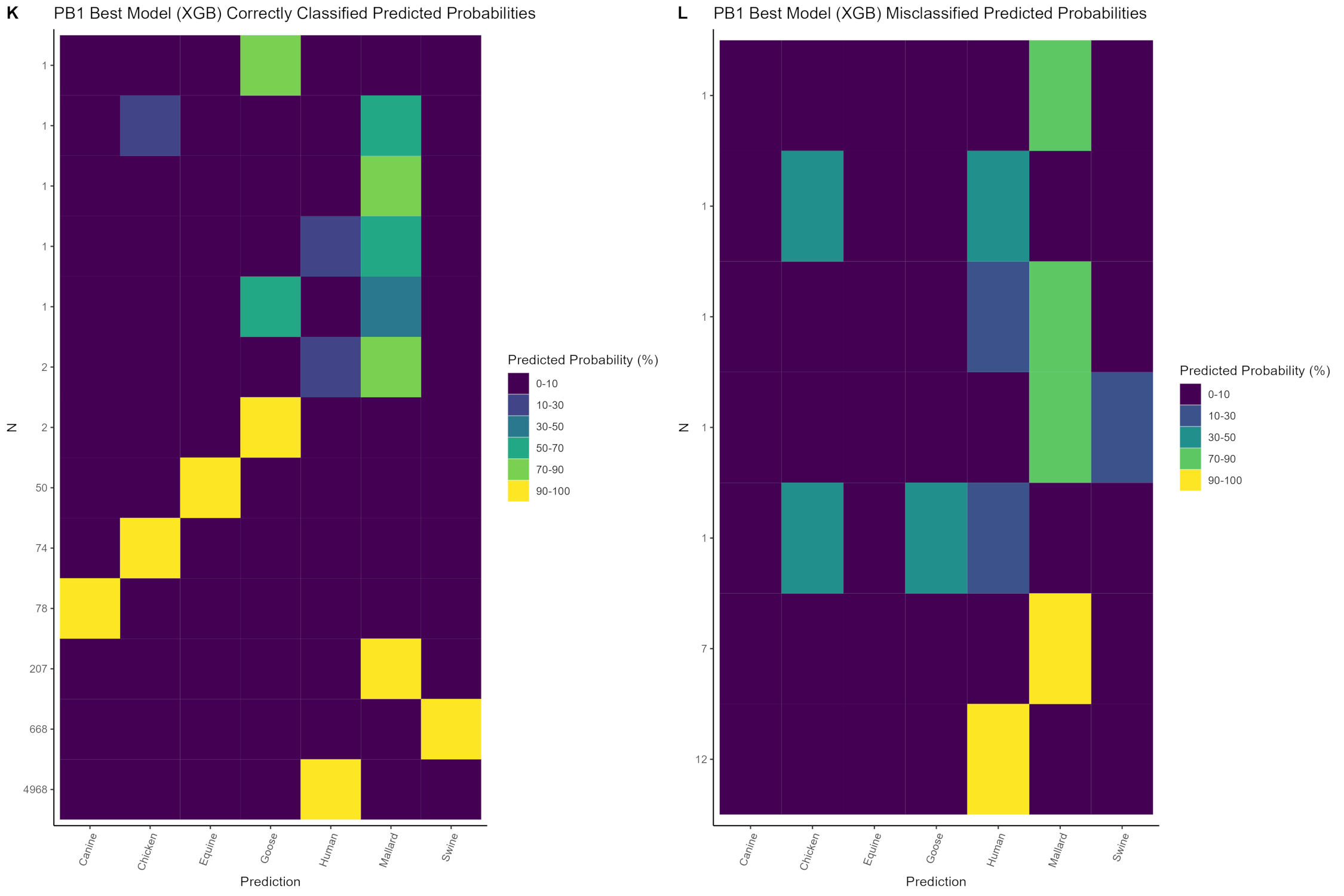

Supplement: S2 Fig — Heatmaps for the PB1 genome segment using the XGBoost model (XGB), A shows the predicted probabilities for the correctly classified sequences and B shows the predicted probabilities for the misclassified sequences. Predicted probabilities are read as rows, and the N on the y axis denotes the number of sequences with the predicted probability pattern shown for the respective row. (TIFF) [file pone.0336142.s011.tiff]

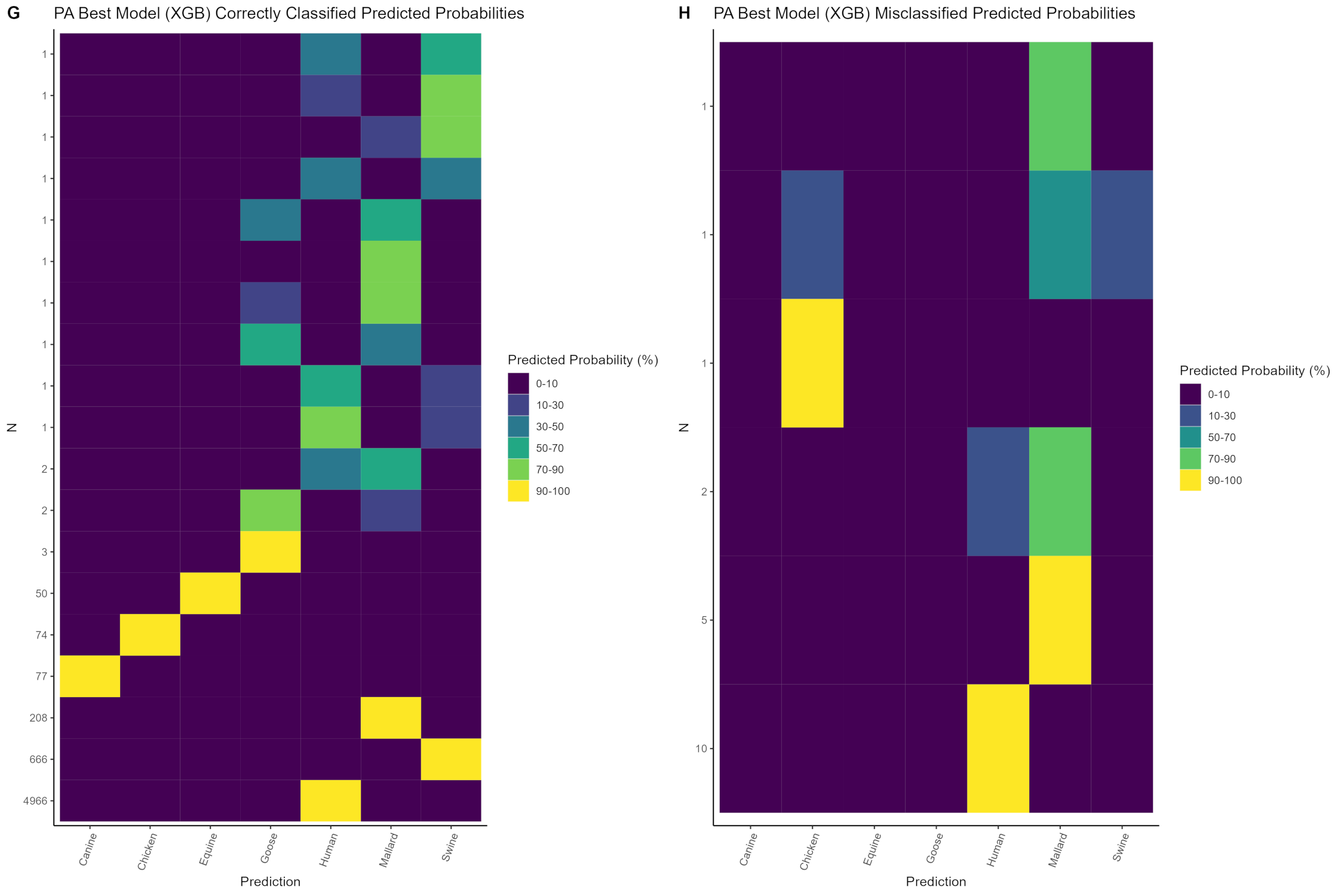

Supplement: S3 Fig — Heatmaps for the PA genome segment using the XGBoost model (XGB), A shows the predicted probabilities for the correctly classified sequences and B shows the predicted probabilities for the misclassified sequences. Predicted probabilities are read as rows, and the N on the y axis denotes the number of sequences with the predicted probability pattern shown for the respective row. (TIFF) [file pone.0336142.s012.tiff]

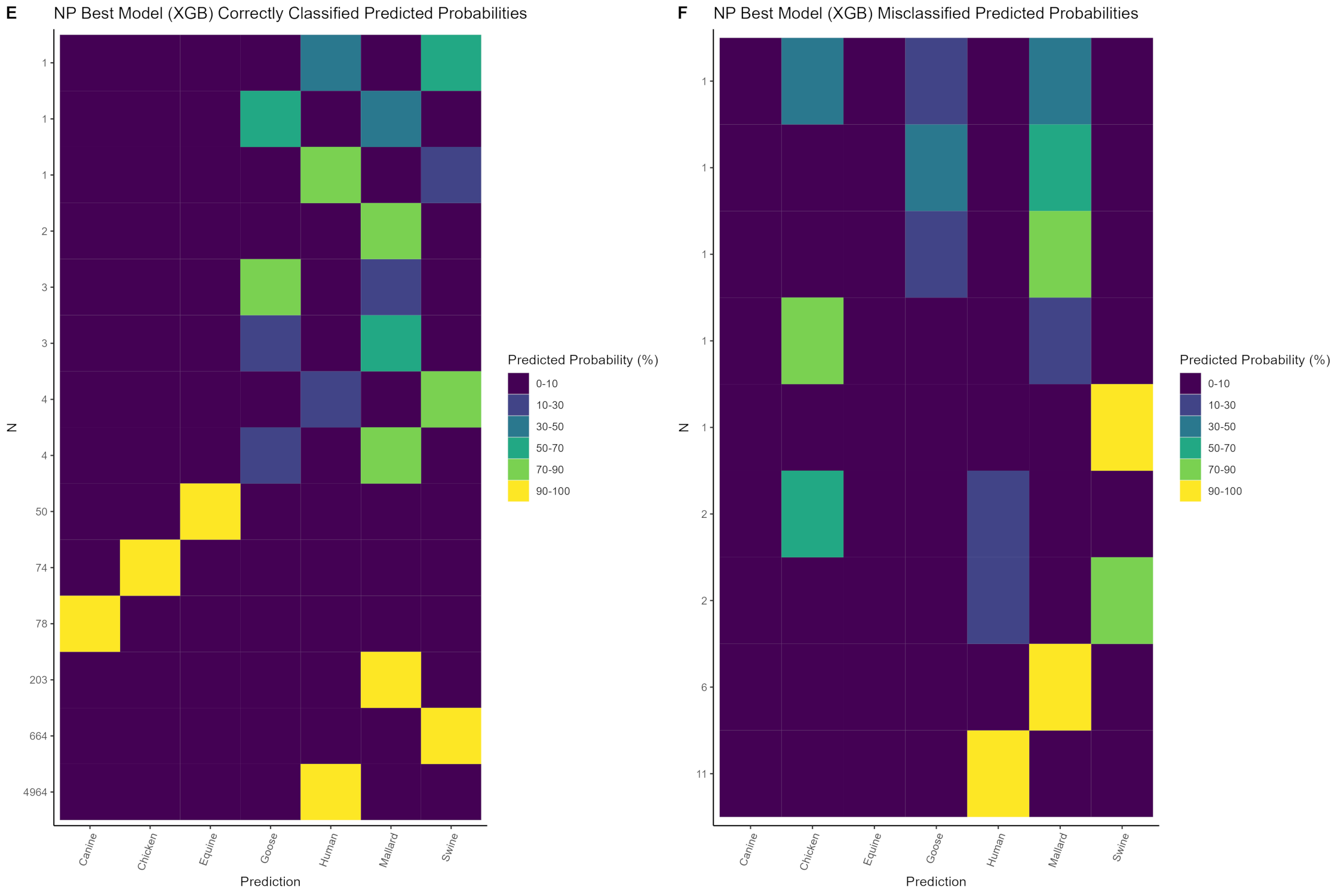

Supplement: S4 Fig — Heatmaps for the NP genome segment using the XGBoost model (XGB), A shows the predicted probabilities for the correctly classified sequences and B shows the predicted probabilities for the misclassified sequences. Predicted probabilities are read as rows, and the N on the y axis denotes the number of sequences with the predicted probability pattern shown for the respective row. (TIFF) [file pone.0336142.s013.tiff]

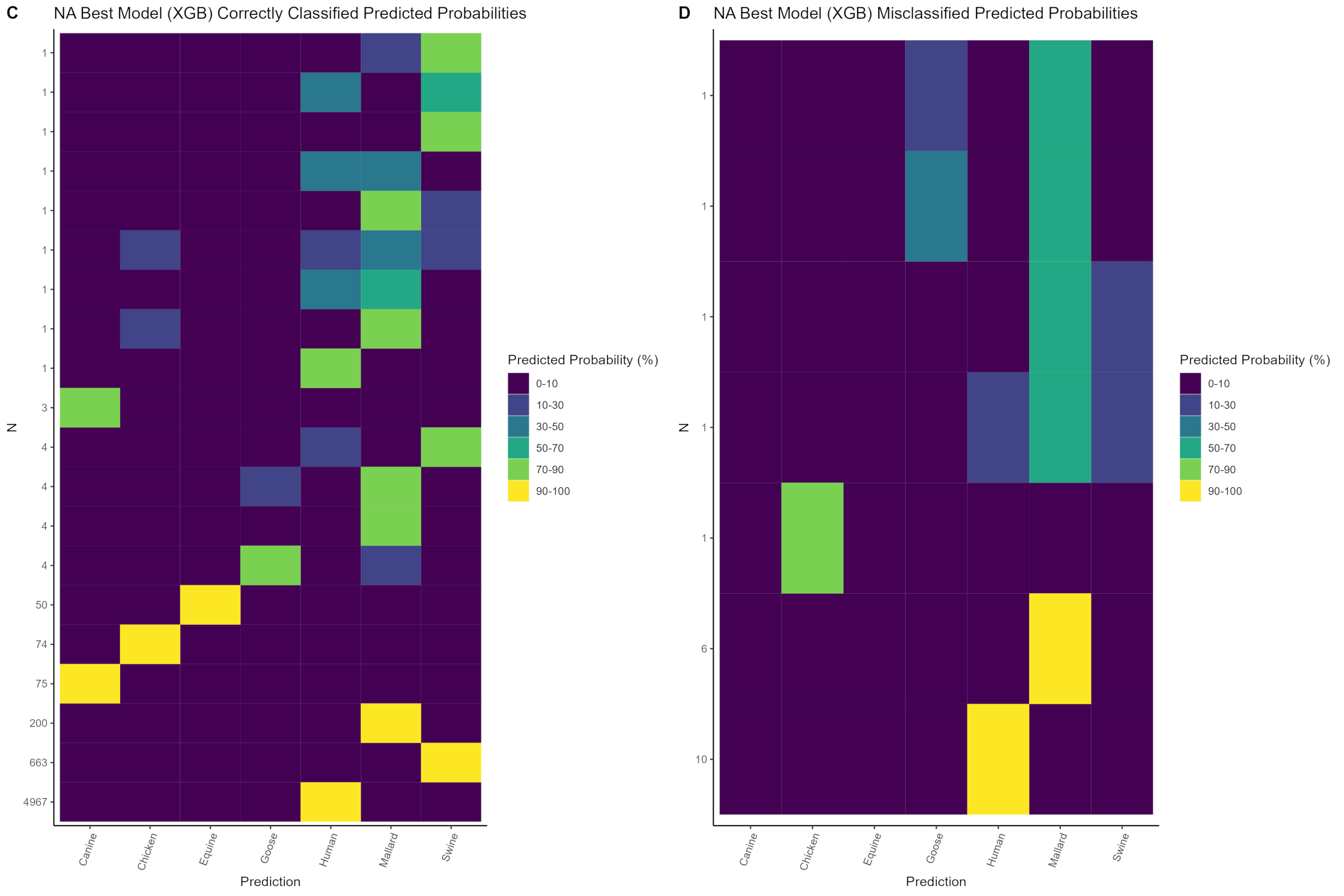

Supplement: S5 Fig — Heatmaps for the NA genome segment using the XGBoost model (XGB), A shows the predicted probabilities for the correctly classified sequences and B shows the predicted probabilities for the misclassified sequences. Predicted probabilities are read as rows, and the N on the y axis denotes the number of sequences with the predicted probability pattern shown for the respective row. (TIFF) [file pone.0336142.s014.tiff]

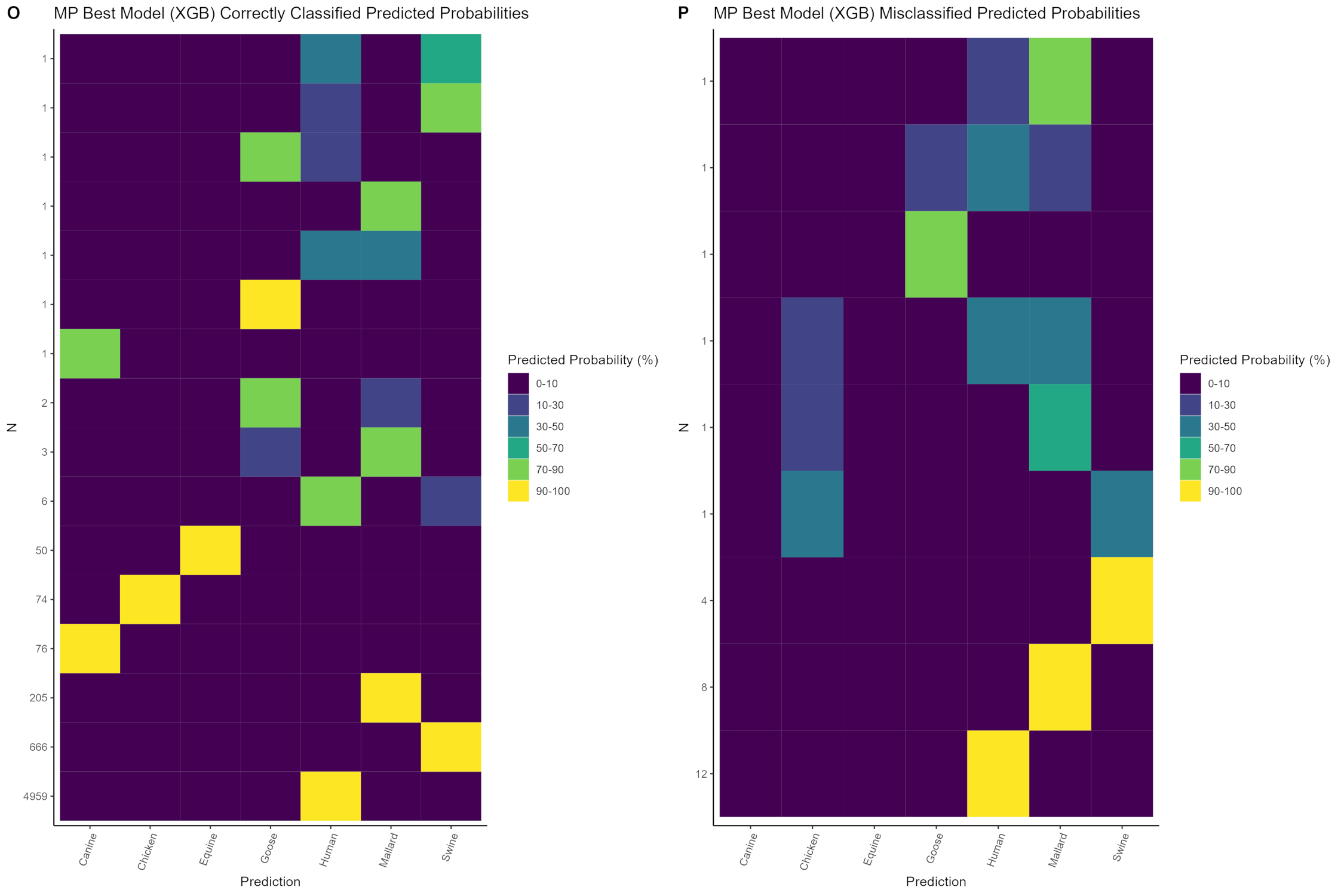

Supplement: S6 Fig — Heatmaps for the MP genome segment using the XGBoost model (XGB), A shows the predicted probabilities for the correctly classified sequences and B shows the predicted probabilities for the misclassified sequences. Predicted probabilities are read as rows, and the N on the y axis denotes the number of sequences with the predicted probability pattern shown for the respective row. (TIFF) [file pone.0336142.s015.tiff]

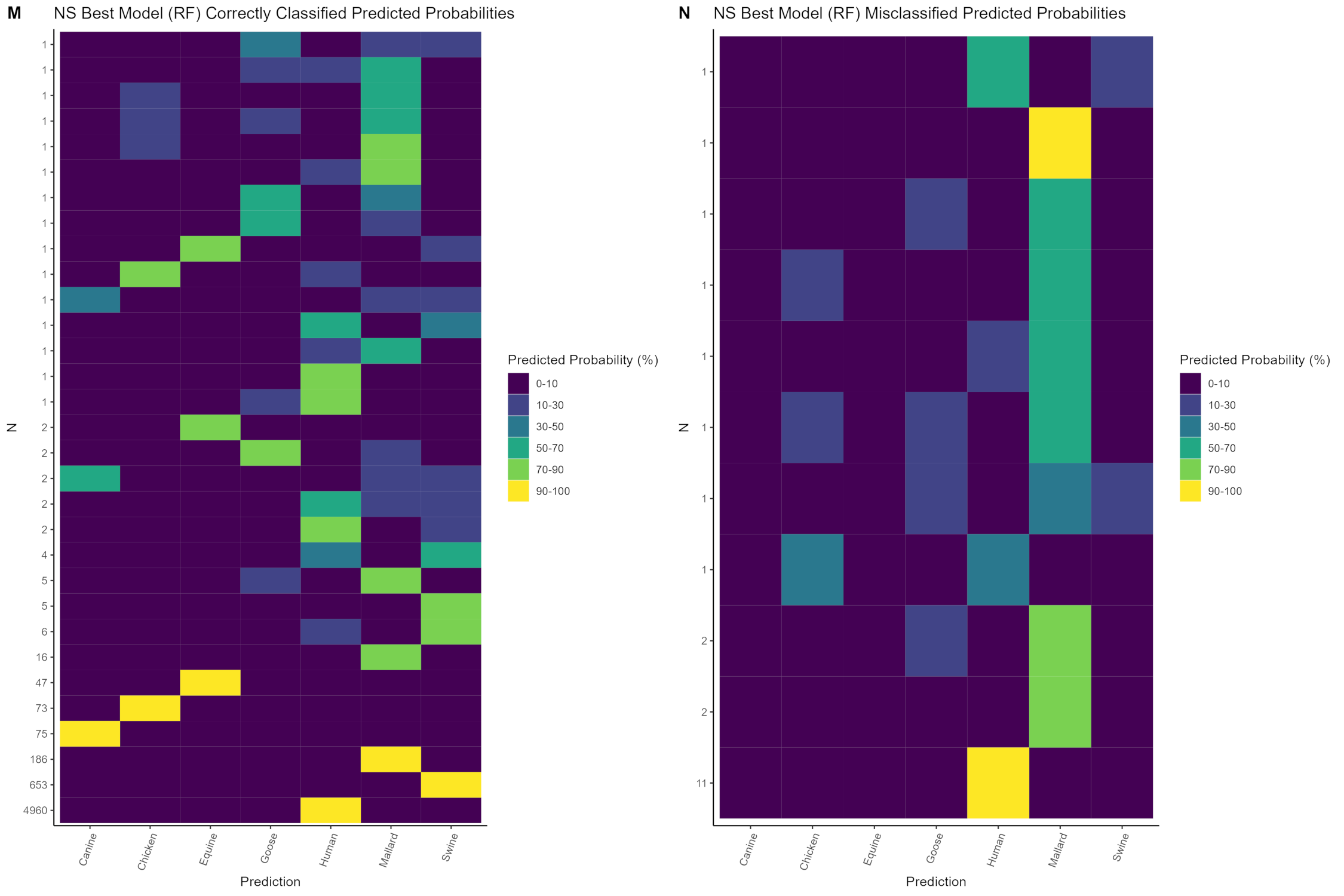

Supplement: S7 Fig — Heatmaps for the NS genome segment using the random forest model (RF), A shows the predicted probabilities for the correctly classified sequences and B shows the predicted probabilities for the misclassified sequences. Predicted probabilities are read as rows, and the N on the y axis denotes the number of sequences with the predicted probability pattern shown for the respective row. (TIFF) [file pone.0336142.s016.tiff]

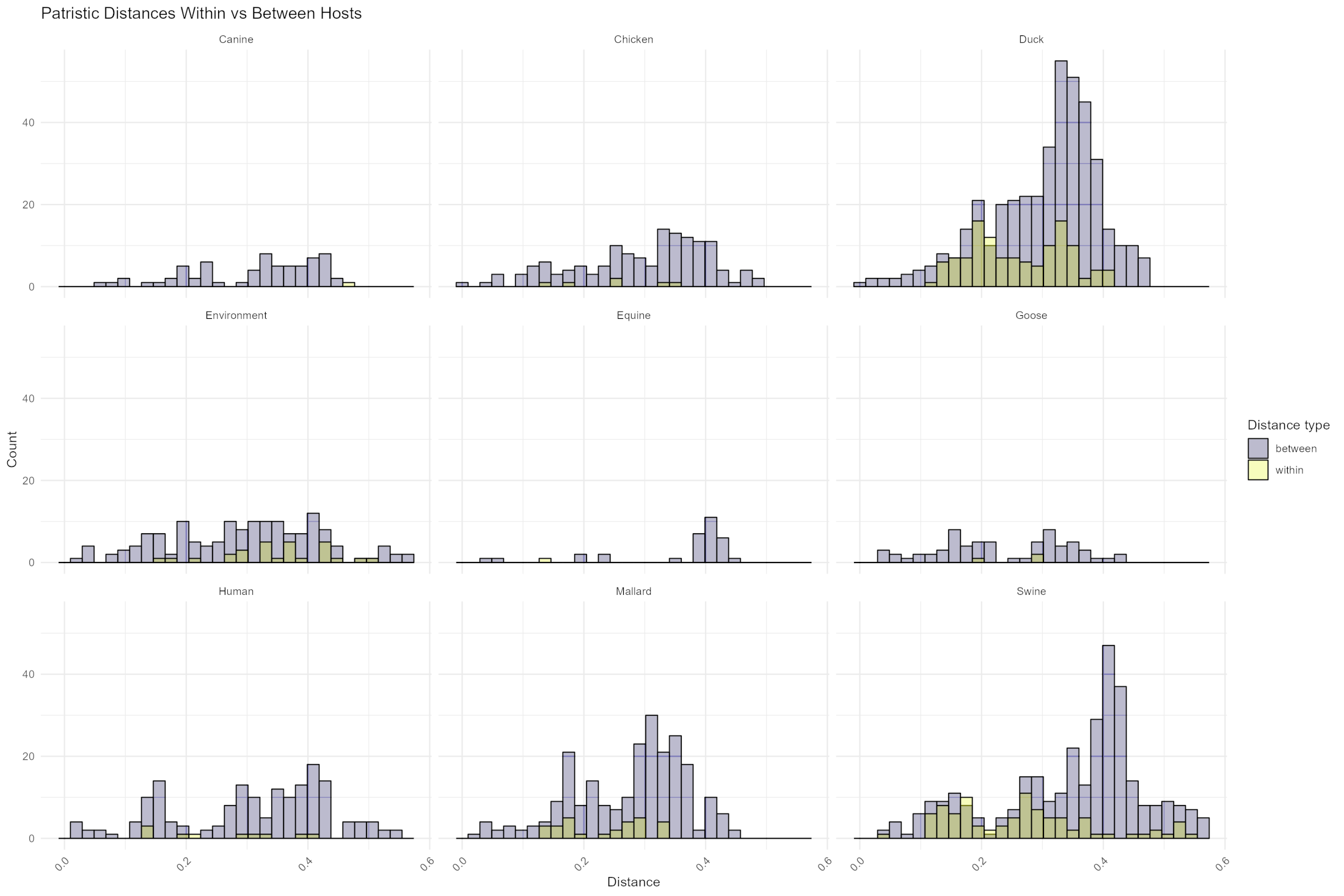

Supplement: S8 Fig — Patristic distances between and within each of the 7 host classes plotted separately. (TIFF) [file pone.0336142.s017.tiff]
