# Supplementary material for: Evaluating machine learning approaches for host prediction using H3 influenza genomic data
Source: PLoS One. 2025 Nov 5;20(11):e0336142. doi: 10.1371/journal.pone.0336142 (PMC12588535; doi:10.1371/journal.pone.0336142)
Supplement: S1 File — (DOCX) [file pone.0336142.s018.docx]

**S1 File. Supplementary information on the sequence selection criteria for each of the databases**

Sequence sets from the IVD were downloaded by selecting the genome set tab with “A” as the chosen type, “H3” as the chosen H subtype, “complete only” as the chosen genome set length, and “any” as the chosen host, country/region, and N subtype. Lab strain sequence sets and sequence sets of mixed subtype were excluded in the additional filters option. Sequence sets from the BV-BRC database were downloaded by selecting the genome search tab with “Influenza A virus” as the chosen pathogen group, “H3N-, H3N1, H3N2, H3N2v, H3N3-H3N9, H3Nx” as the chosen subtypes, and “complete” as the chosen genome length. Sequence sets from the Epiflu database were downloaded by selecting “A” as the chosen type, “H3” as the chosen H subtype, “PB2, PB1, PA, HA, NP, NA, MP, NS” as the chosen required segments, “only complete” as the chosen genome length, and “-all-“ as the chosen host, location, and N subtype.
