# Supplementary material for: Evaluating machine learning approaches for host prediction using H3 influenza genomic data
Source: PLoS One. 2025 Nov 5;20(11):e0336142. doi: 10.1371/journal.pone.0336142 (PMC12588535; doi:10.1371/journal.pone.0336142)
Supplement: S2 File — (DOCX) [file pone.0336142.s019.docx]

**S2 File. Supplementary information on the Fig 1A and 1B heatmap predicted probabilities**

The correctly classified sequences for Pattern 1 (70-90% mallard and classes with less than 10% predicted probabilities) were identified with the accession numbers and strains of GQ240821 A/mallard/Hungary/19616/2007 (H3N8), MN530412 A/mallard/South Korea/N06-1355/2006 (H3N2) and MN988126 A/mallard/Alaska/AK18-WB1-048B/2018 (H3N8), respectively. Sequence GQ240821 had predicted probabilities of 89% mallard, 7.2% goose, 2.1% human, and 1.2% chicken. Sequence MN530412 had predicted probabilities of 87% mallard, 8.3% chicken, 1.4% canine, 1.3% goose, 0.9% human, 0.9% swine, and 0.4% equine. Sequence MN988126 had predicted probabilities of 81.8% mallard, 9.6% goose, 5.2% chicken, 2% human, 0.7% swine, 0.4% equine, and 0.4% canine. Sequence GQ240821 was investigated in the discussion section of the manuscript. Sequence MN530412 was found to cluster with Korean domestic ducks (Youk et al., 2020). Sequence MN988126 did not have available literature to investigate.

The correctly classified sequences for Pattern 2 (70-90% goose and 10-30% mallard) were identified with the accession numbers and strains of AB569511 A/goose/Zambia/06/2008 (H3N8), KY131297 A/emperor goose/Alaska/UGAI15-6734/2015 (H3N8) and KX949476 A/emperor goose/Alaska/UGAI15-6759/2015 (H3N8), respectively. Sequence AB569511 had predicted probabilities of 85.3% goose and 13% mallard. Sequence KY131297 and KX949476 both had predicted probabilities of 86% goose and 11.5% mallard. Sequence AB569511 was investigated in the discussion section of the manuscript. Sequences KY131297 and KX949476 did not have available literature to investigate.

The correctly classified sequences for Pattern 3 (70-90% swine and 10-30% human) were identified with the accession numbers and strains of CY116315 A/swine/England/87842/1990 (H3N2), KM028055 A/swine/Guangxi/2803/2011 (H3N2), EU798789 A/swine/Korea/CAS05/2004, EU798795 A/swine/Korea/CY07/2007 (H3N2), MW848635 A/swine/Spain/46314-2/2020 (H3N1), respectively. Sequence CY116315 had predicted probabilities of 82.2% swine and 14.8% human. Sequence KM028055 had predicted probabilities of 87% swine and 13% human. Sequence EU798789 had predicted probabilities of 82.7% swine and 16.7% human. Sequence EU798795 had predicted probabilities of 85.2% swine and 14.7% human. Sequence MW848635 had predicted probabilities of 81.2% swine and 17.7% human. Sequence CY116315 was investigated in the discussion section of the manuscript. Sequence KM028055 was found to cluster with swine sequences with segments 1,3,5,7 and 8 classified into the pdm/09 lineage and segment 2 classified into the triple-reassortant lineage (Liang et al., 2014). Sequence EU798789 was found to cluster with human sequences from New York and appeared to form a novel cluster (Pascua et al., 2008). Additionally, Pascua et al., (2008) also found sequence EU798795 to cluster with swine sequences from the United States. Sequence MW848635 had no available literature to investigate.

The misclassified sequences for Pattern 4 (90-100% mallard) were identified with the accession numbers and strains of KU158890 A/chicken/Nanjing/B854-2/2011(H3N8), HQ165996 A/chicken/Pakistan/NARC-16945/2010 (H3N1), JX080755 A/greater white-fronted goose/Alaska/44064-108/2006 (H3N2), JX080757 A/greater white-fronted goose/Alaska/44299-002/2007 (H3N8), CY138153 A/snow goose/Nunavet/03438/2010 (H3N8), and KJ889415 A/swine/Kazakhstan/106/1985 (H3N6). Sequence KU158890 had predicted probabilities of 91% mallard, 2.8% human, 1.6% goose, 1.5% chicken, 1.5% canine, and 1% swine. Sequence HQ165996 had predicted probabilities of 99.1% mallard, 0.3% goose, 0.2% swine, and 0.1% canine, chicken, equine, and human. Sequence JX080755 had predicted probabilities of 99.5% mallard, 0.2% goose, and 0.1% human and chicken. Sequence JX080757 had predicted probabilities of 97.4% mallard, 1.5% goose, and 0.2% canine, chicken, equine, human, and swine. Sequence CY138153 had predicted probabilities of 99.7% mallard and 0.1% goose and swine. Sequence KJ889415 had predicted probabilities of 97.4% mallard, 1.6% goose, 0.4% swine, 0.2% human and chicken, and 0.1% canine and equine. Sequence KU158890 was investigated in the discussion section of the manuscript. Sequence HQ165996 was found to cluster with duck and mallard H3N8 strains (Siddique et al., 2012). Sequences JX080755 and JX080757 were sampled from the Yukon-Kushokwim Delta which was identified as a major migratory flyway between different avian species (Reeves et al., 2013). Sequence KJ889415 was investigated alongside European avian-like swine influenza viruses (Krumbholz et al., 2014). Sequence CY138153 had no available literature for investigation.

The misclassified sequences for Pattern 5 (90-100% human) were identified with the accession numbers and strains of LC644998 A/swine/Zambia/51/2018 (H3N2), KC197821 A/swine/Colombo/48/2004 (H3N2), FJ157986 A/swine/Guangxi/1/2004 (H3N2), MW911600 A/swine/Guatemala/MM-160/2013 (H3N2), MN608553 A/swine/Michigan/A02478737/2019 (H3N2), OQ588211 A/swine/Ohio/OH19-15895/2019 (H3N2), OQ588491 A/swine/Ohio/OH19-7727-1/2019 (H3N2), OQ588499 A/swine/Ohio/OH19-7727-2/2019 (H3N2), CY158745 A/swine/Saskatchewan/02903/2009 (H3N2), MN436839 A/swine/Virginia/A02478581/2019 (H3N2), CY009308 A/swine/Wisconsin/194/1980 (H3N2). Sequence LC644998 had predicted probability of 100% human. Sequence KC197821 had predicted probability of 99.9% human. Sequences FJ157986 and CY009308 had predicted probability of 99.8% human and 0.01% swine and canine. Sequence MW911600 had predicted probability of 99.9% human. Sequences MN608553 and OQ588211 had predicted probability of 99.6% human and 0.4% swine. Sequences OQ588491, OQ588499, and MN436839 had predicted probability of 99.5% human and 0.4% swine. Sequence CY158745 had predicted probability of 99.3% human and 0.5% swine. Sequence LC644998 was investigated in the discussion section of the manuscript. Sequence KC197821 was found to cluster with human strains (Perera et al., 2013). The remaining 9 sequences had no available literature for investigation.

The misclassified sequence for Pattern 6 (split between mallard, goose, and classes with less than 10%) was identified with accession number and strain of JX080759 A/greater white-fronted goose/Alaska/44299-055/2007. Sequence JX080759 had predicted probabilities of 47.4% mallard, 37.5% goose, 8.1% chicken, 2.3% human, 1.8% swine, 1.6% equine, and 1.3% canine. Sequence JX080759 was investigated in the discussion section of the manuscript.

The misclassified sequences for Pattern 7 (70-90% mallard and classes with less than 10% predicted probabilities) were identified with the accession numbers and strains JX096504 A/swine/Guangdong/L21/2011 (H3N2) and KM222548 A/chicken/Shanghai/LPM2/2013 (H3N2), respectively. Sequence JX096504 had predicted probabilities of 81.6% mallard, 6.6% chicken, 5.8% human, 1.6% swine, 1% canine, and 1% equine. Sequence KM222548 had predicted probabilities of 85.9% mallard, 8.3% human, 1.7% chicken, 1.5% goose, and 1.4% canine. Sequence JX096504 was investigated in the discussion section of the manuscript. Sequence KM222548 was found to cluster with domestic poultry sequences and was sampled from a slaughterhouse which housed domestic poultry in close proximity, providing ample opportunities for between-species transmission (Yang et al., 2015).

**References**

Krumbholz, A., Lange, J., Sauerbrei, A., Groth, M., Platzer, M., Kanrai, P., Pleschka, S., Scholtissek, C., Büttner, M., Dürrwald, R., & Zell, R. (2014). Origin of the European avian-like swine influenza viruses. *The Journal of general virology*, *95*(Pt 11), 2372–2376. https://doi.org/10.1099/vir.0.068569-0

Liang, H., Lam, T. T., Fan, X., Chen, X., Zeng, Y., Zhou, J., Duan, L., Tse, M., Chan, C. H., Li, L., Leung, T. Y., Yip, C. H., Cheung, C. L., Zhou, B., Smith, D. K., Poon, L. L., Peiris, M., Guan, Y., & Zhu, H. (2014). Expansion of genotypic diversity and establishment of 2009 H1N1 pandemic-origin internal genes in pigs in China. *Journal of virology*, *88*(18), 10864–10874. <https://doi.org/10.1128/JVI.01327-14>

Pascua, P. N., Song, M. S., Lee, J. H., Choi, H. W., Han, J. H., Kim, J. H., Yoo, G. J., Kim, C. J., & Choi, Y. K. (2008). Seroprevalence and genetic evolutions of swine influenza viruses under vaccination pressure in Korean swine herds. *Virus research*, *138*(1-2), 43–49. <https://doi.org/10.1016/j.virusres.2008.08.005>

Perera, H. K., Wickramasinghe, G., Cheung, C. L., Nishiura, H., Smith, D. K., Poon, L. L., Perera, A. K., Ma, S. K., Sunil-Chandra, N. P., Guan, Y., & Peiris, J. S. (2013). Swine influenza in Sri Lanka. *Emerging infectious diseases*, *19*(3), 481–484. https://doi.org/10.3201/eid1903.120945

Reeves, A. B., Pearce, J. M., Ramey, A. M., Ely, C. R., Schmutz, J. A., Flint, P. L., Derksen, D. V., Ip, H. S., & Trust, K. A. (2013). Genomic analysis of avian influenza viruses from waterfowl in western Alaska, USA. *Journal of wildlife diseases*, *49*(3), 600–610. https://doi.org/10.7589/2012-04-108

Siddique, N., Naeem, K., Ahmed, Z., Abbas, M. A., Farooq, S., & Malik, S. A. (2012). Isolation, identification, and phylogenetic analysis of reassortant low-pathogenic avian influenza virus H3N1 from Pakistan. *Poultry science*, *91*(1), 129–138. https://doi.org/10.3382/ps.2011-01530

Yang, D., Liu, J., Ju, H., Ge, F., Wang, J., Li, X., Zhou, J., & Liu, P. (2015). Genetic analysis of H3N2 avian influenza viruses isolated from live poultry markets and poultry slaughterhouses in Shanghai, China in 2013. *Virus genes*, *51*(1), 25–32. https://doi.org/10.1007/s11262-015-1198-5

Youk, S. S., Lee, D. H., Jeong, J. H., Pantin-Jackwood, M. J., Song, C. S., & Swayne, D. E. (2020). Live bird markets as evolutionary epicentres of H9N2 low pathogenicity avian influenza viruses in Korea. *Emerging microbes & infections*, *9*(1), 616–627. <https://doi.org/10.1080/22221751.2020.1738903>
