# Supplementary material for: Evaluating machine learning approaches for host prediction using H3 influenza genomic data
Source: PLoS One. 2025 Nov 5;20(11):e0336142. doi: 10.1371/journal.pone.0336142 (PMC12588535; doi:10.1371/journal.pone.0336142)
Supplement: S3 File — (DOCX) [file pone.0336142.s020.docx]

**S3 File. Supplementary information on the environment case study**

Predicted probability plots for case study 4 identified that a large majority of environment sequence sets were being predicted as mallard with high predicted probabilities in addition to a small group of sequence sets being predicted as swine with high predicted probabilities across all genome segments. Phylogenetic analysis using our maximum-likelihood tree somewhat supported these model predictions as the representative environment HA sequences were found to cluster primarily with mallard and duck HA representative sequences, with a few clusters with swine, human, or goose HA representative sequences. No further information through the literature was available for the sequence sets being predicted as mallard aside from a majority of them originating from Maryland, United States, however, metagenomics is an approach that has gained traction over time for investigation and surveillance of IAV in waterfowls and avians as a whole (Ahrens et al., 2023; K. Kuchinski et al., 2022; K. S. Kuchinski et al., 2024). The reasoning behind this approach is that it is easier to collect sequences from the environment without disrupting wildlife compared to traditional methods and that IAV strains from both common and rare avian species will naturally accumulate in wild avian habitats (K. S. Kuchinski et al., 2024). Accordingly, it is reasonable to assume that a majority of environment sequences were sampled from wild waterfowl and avian habitats, hence the mallard predictions. For the sequence sets being predicted as swine, 6 were consistently predicted as swine across all segments. These sequence sets consisted of the strains A/environment/Indiana/15TOSU25204/2015,  A/environment/Indiana/15TOSU25207/2015,  A/environment/Indiana/16TOSU3545/2016,  A/environment/Indiana/16TOSU3553/2016,  A/environment/Indiana/16TOSU3767/2016, and  A/environment/Indiana/16TOSU3854/2016. A prior study had also predicted these sequences as the swine class, although for only the HA segment (Alberts et al., 2024). Four of these sequence sets were found to originate from agricultural fairs involving swine in Indiana, United States (Lauterbach et al., 2018), with the remaining two labelled as from a livestock exhibition wipe in Indiana, United States, supporting the predicted probabilities for these environment sequence sets as swine.

**References**

Ahrens, A. K., Selinka, H.-C., Wylezich, C., Wonnemann, H., Sindt, O., Hellmer, H. H., Pfaff, F., Höper, D., Mettenleiter, T. C., Beer, M., & Harder, T. C. (2023). Investigating Environmental Matrices for Use in Avian Influenza Virus Surveillance-Surface Water, Sediments, and Avian Fecal Samples. Microbiology Spectrum, 11(2). https://doi.org/10.1128/SPECTRUM.02664-22

Alberts, F., Berke, O., Maboni, G., Petukhova, T., & Poljak, Z. (2024). Utilizing machine learning and hemagglutinin sequences to identify likely hosts of influenza H3Nx viruses. Preventive Veterinary Medicine, 233, 106351. https://doi.org/10.1016/J.PREVETMED.2024.106351

Kuchinski, K., Duan, J., Coombe, M., Himsworth, C., Hsiao, W., & Prystajecky, N. (2022). Recovering influenza genomes from wild bird habitats for outbreak prevention and pandemic preparedness. International Journal of Infectious Diseases, 116, S104. https://doi.org/10.1016/J.IJID.2021.12.245

Kuchinski, K. S., Coombe, M., Mansour, S. C., Cortez, G. A. P., Kalhor, M., Himsworth, C. G., & Prystajecky, N. A. (2024). Targeted genomic sequencing of avian influenza viruses in wetland sediment from wild bird habitats. Applied and Environmental Microbiology, 90(2). <https://doi.org/10.1128/AEM.00842-23/SUPPL_FILE/AEM.00842-23-S0007.PDF>

Lauterbach, S. E., Wright, C. M., Zentkovich, M. M., Nelson, S. W., Lorbach, J. N., Bliss, N. T., Nolting, J. M., Pierson, R. M., King, M. D., & Bowman, A. S. (2018). Detection of influenza A virus from agricultural fair environment: Air and surfaces. *Preventive veterinary medicine*, *153*, 24–29. https://doi.org/10.1016/j.prevetmed.2018.02.019
